# Deep Learning-Empowered Digital Twin Using Acoustic Signal for Welding Quality Inspection

**DOI:** 10.3390/s23052643

**Published:** 2023-02-28

**Authors:** Tao Ji, Norzalilah Mohamad Nor

**Affiliations:** School of Mechanical Engineering, Universiti Sains Malaysia, Nibong Tebal 14300, Pulau Pinang, Malaysia

**Keywords:** digital twin, welding site inspection, acoustic signal, deep learning

## Abstract

Weld site inspection is a research area of interest in the manufacturing industry. In this study, a digital twin system for welding robots to examine various weld flaws that might happen during welding using the acoustics of the weld site is presented. Additionally, a wavelet filtering technique is implemented to remove the acoustic signal originating from machine noise. Then, an SeCNN-LSTM model is applied to recognize and categorize weld acoustic signals according to the traits of strong acoustic signal time sequences. The model verification accuracy was found to be 91%. In addition, using numerous indicators, the model was compared with seven other models, namely, CNN-SVM, CNN-LSTM, CNN-GRU, BiLSTM, GRU, CNN-BiLSTM, and LSTM. A deep learning model, and acoustic signal filtering and preprocessing techniques are integrated into the proposed digital twin system. The goal of this work was to propose a systematic on-site weld flaw detection approach encompassing data processing, system modeling, and identification methods. In addition, our proposed method could serve as a resource for pertinent research.

## 1. Introduction

Several countries have selected Industry 4.0 as a strategic goal for their industrial development because of its huge potential and exciting possibilities. In industrial production, welding is the most common method for joining materials. Various welding processes can be accomplished very efficiently using automatic equipment, such as industrial robots [1]. Providing the robot with only expected instructions allows it to begin working immediately.

Although industrial robots have a high level of intelligence, the whole welding process does not have the same level of intelligence. Online intelligent weld defect detection still has a troublesome track record, at least when it comes to detecting weld defects [2]. Online weld testing not only makes weld quality more reliable and stable, but also improves production efficiency and reduces production costs [3]. GMAW (Gas Metal Arc Welding) is one of the most commonly used welding processes. However, high temperature, spatter, high-strength arc light, and complex welding environments make GMAW online weld detection difficult [4].

The most popular method for finding weld defects online is to use visual images to obtain more understandable weld and weld pool information. By gathering an image of the weld center and by examining the geometric and spatial distribution properties of the weld, Ma suggested a weld defect detection method based on active visual sensing and machine learning [5].

Zhang developed an image collection system to acquire image data in all welding directions. After that, a CNN (Convolutional Neural Network) is used to identify it. It is important to note that the system incorporates welding arc light as complementary information rather than weakening or eliminating arc light interference [6]. Arc changes can be reflected in welding current and voltage. In order to determine the weld penetration, Cao used the arc voltage in the peak current period and the average arc voltage during this time and proposed a bilinear model to design a gain-scheduled model predictive controller [7]. Ultrasonic testing has also been used for online testing as a conventional, non-destructive procedure. Bonikila established a non-destructive weld detection method based on ultrasonics utilizing a machine learning algorithm [8].

The analysis of arc acoustic signals has always been a crucial factor in the online assessment of weld quality. An acoustic signal may perfectly match the detection criteria, especially in the online detection of weld fields. However, acoustic signals are easily interrupted, and welding is not a linear change process. A reason for this is the fact that acoustic signal extraction and analysis are highly challenging.

Through experimentation, Horvat examined the acoustic signal of GMAW welding, identified the two primary noise sources, and proposed a classification algorithm [9]. Gao developed the notion that professional welders can detect welding process flaws through their auditory experience. By gathering significant crowd input, a subjective assessment model based on human hearing was presented [10]. Following the extraction of acoustic information using LPCC (Licensed Professional Clinical Counselors), Lv suggested a BPANN (Back Propagation Artificial Neural Network) pie-change algorithm and constructed a controller using this algorithm. The controller accuracy in the experiment ranged from 80% to 90% [11]. Cui studied the arc bias blowing phenomenon in K-TIG (Keyhole Tungsten Inert Gas) welding and subsequently suggested an ECOC-SVM-GSCV technique based on machine learning to categorize weld penetration flaws [3]. Liu focused on cold metal transfer lap welding (CMT) and emphasized how air intake affects the caliber of the weld. An RNN-based BiLSTM-CTC method was suggested to determine the signal features by tracking the sound signal of air intake [12]. Acoustic experimental hardware was upgraded by Chen, who also set up an array of several microphones and used the FastICA (Fast algorithm for Independent Component Analysis) to separate weld noise [13]. Wang developed a central audit perception model, tested the model effectiveness in online weld detection, and used the model to examine the human hearing principle to identify defects in GMAW welding [14].

The acoustic signal is a key parameter in welding site inspection. The real-time and accurate acoustic diagnosis of weld defects has long been a challenging subject because of the complicated on-site environment and highly non-linear welding process. In order to identify weld defects, this study suggests a system based on digital twin acoustic signals. Real-time, accurate defect identification was considered when defining the fundamental needs of the digital twin system.

This paper presents a digital twin system for weld sound detection that includes the setup of hardware for experimental purposes, creating a virtual environment for experimentation. In Section 2, the communication network of the virtual reality system and other components are introduced. In Section 3, the preprocessing approach for sound signals is introduced. By examining the time-domain and frequency-domain properties of the signal, we then suggest an adaptive wavelet threshold denoising method. To extract signal features and to detect and classify signals based on the timing properties of acoustic signals and the requirements of real-time systems, an improved SeCNN-LSTM deep learning model is proposed in Section 4. In this work, seven other models were tested and compared with the SeCNN-LSTM model, and a variety of metrics, such as precision and sample balance, were used for validation. This paper is summarized in Section 5, which also examines its flaws and suggests a future line of inquiry.

## 2. Digital Twin System

### 2.1. Digital Twin of Industrial Robot

Digital twin (DT) is currently acknowledged as one of the key avenues for advancement in achieving Industry 4.0 and industrial intelligence [15]. The earliest application of DTs was in the aerospace industry to build a fictitious digital object for tracking and forecasting spacecraft performance [16]. Digital twins have started to show their potential in recent years as a result of the significant advancement in and richness of the communication sector as well as the precision and variety of sensors. DTs have swiftly spread across numerous sectors and research fields in just a few years [17]. DTs represent a potent tool for producing real-time analysis systems, because they are virtual replicas of real systems and can communicate in two directions with the physical copy [18].

Lu proposed a straightforward DT model in their article [19]. The model makes it clear that the inclusion of physical objects, digital objects, and communication is the fundamental characteristic of DTs. Information models and data processing operations make up the majority of digital objects. Real-time, bidirectional communication is the fundamental criterion. Ren developed a digital twin of a coaxial single-sided resistance spot weld for real-time prediction of transient temperature fields, based on an experimentally validated finite element model [20].

Numerous reports have been published so far on the use of DTs in the realm of industrial robotics. Xu presented an industrial cloud robot based on DT that combines cloud computing and industrial robots. They utilized it for sorting logistics [21]. With the use of a DT system, Zhuang assembled and predicted spaceships using a model of a workshop complete with industrial robots [22]. Wang focused on the picture data of the weld pool during the welding operation in order to construct a DT system [23]. Tiparey suggested a way for flexible industrial robots to pick and place workpieces based on the idea of DT [24].

The real-time reflection of the condition of physical things and bidirectional communication are the fundamental requirements of DTs. The approach to on-site weld inspection is entirely consistent with this. Tao, F proposes a digital twin modelling approach for five-dimensional models [25], as shown in Formula (1).The DT system used in this research utilizes the five-dimensional model creation method.

(1) MDT=(PE,DE,Ss,DD,CN)

where PE is the physical object, DE is the digital object, Ss is the service target or service standard, DD is data processing, and CN is the communication mode.

#### 2.1.1. Physical Entity

The physical entity is the physical component of the activity or the component that needs to be digitized. The physical parts of industrial robots usually include the robot body, the control cabinet, and FlexPendant. Welding robots also need to include gas cylinders and welding control cabinets. There are numerous different structures in the robot body. The robot model used in this paper was KUKA KR 10 R1420 HP, which is a classic six-axis serial robot. The robot controller model was KUKA KR C4. In addition, there were supporting wire feeding mechanisms, welding gun, and workpieces. A TBi Industries RoboMIG RM2 system was adopted for welding gun and wire feeder. The microphone and acoustic signal acquisition tools employed is KY-037, which consists of three main components: microphone; audio amplifier and comparator. An electret condenser microphone (ECM) as an audio sensor to detect and measure the area physically sounds and produce an analog signal. The audio amplifier receives the analogue signal from the ECM and amplifies it. The comparator compares the amplified signal with the reference value and change output level. The physical entity also includes the workspace where the robot is located, which was an enclosed space of about 10 m^2^ in our case. Computing resources are I7 CPU and RTX 3050Ti GPU.

#### 2.1.2. Digital Entity

The digital entity is a highly integrated and quantified model in the DT system. It accurately simulates a wide range of physical entity features. The digital entity characteristics are displayed in Formula (2).

(2) DE=(Gm,Pm,Dm,Rm)

where Gm is a geometric model. The geometric model of an industrial robot is usually represented by its 3D model, which was here built using CAD software. It should be noted that the geometric model of the workpiece also had to be built, because the focus of this paper is the robot welding procedure.

In the above equation, Pm is the physical property model. Through the physical property model, the material and physical properties of the entity are expressed in the physical property model. Welding wire materials and their physical qualities, workpiece materials and their welding characteristics, and welding shielding gas are all important considerations in welding process experiments. The characteristics of the materials used here are shown in Table 1.

In the above equation, Dm is a dynamic model that mostly refers to the robot trajectory, drive mode, and other kinematic and power information. The geometry of the workpiece and the operator, in general, determine the motion information of an industrial robot.

In the above equation, Rm is the rule model. The secret to unlocking the intelligence of the digital twin system is the rule model. It refers to the guidelines for robot evaluation, prediction, and judgment. A sort of SeCNN-LSTM deep learning algorithm was employed in this experiment to develop the rule model for detecting welding errors in robots, which are crucial to locating weld flaws. In Section 3 and Section 4, this section is thoroughly explained.

#### 2.1.3. Service Project

Ss refers to the goal of common services of digital entities and physical entities and is expressed by Formula (3).

(3) Ss=(Fun,Input,Output,Qua,St)

where Fun is a functional term. This experiment’s primary purpose was to identify welding process defects by means of the on-the-job monitoring of weld defects. Robot motion and position data made up the majority of Input and Output, with the sound signals captured by sensors also serving as inputs. Qua refers to quality, which is understood as weld quality and recognition efficiency in this paper. Presenting comparative tests, Section 5 of this paper thoroughly introduces the topic of recognition efficiency. St is the robot running condition.

#### 2.1.4. Data

Since the DT system is essentially a virtual picture product, it must receive data from every system participant.

(4) DD=(DPe,DDe,DSs,De)

where DPe are data from robots and sensors, DDe are the feedback data of virtual entities, DSs are server data, and De are the expert data in the knowledge base.

#### 2.1.5. Communication

The DT system uses a variety of communication channels because of its stringent requirements for information transmission. BiDE_DD, BiDE_Ss, and BiDD_Ss stand for digital entities and databases, database and server interaction, and two-way communication between the digital entity and the server, respectively. The communication between the physical entity and the server is represented by PE_DD and PE_Ss, and by PE_DD and PE_Ss, respectively. It is important to note that the industrial robot is highly integrated equipment. Typically, the industrial robot control cabinet or FlexPendant has a few external connectors. Some degree of permission to control the motion status of each robot joint can be obtained through the external interface. BiINrob refers to two-way external interface communication with the robot. On the KUKA robot control cabinet and FlexPendant we employed, there were Ethernet external ports. Since EXsen was used to connect external sound sensors, only one-way communication was possible in this section.

(5) $$\begin{array}{cc} CN= & (PE\_DD,BiDE\_DD,PE\_Ss,BiDE\_Ss,\\ \; & BiDD\_Ss) \end{array}$$

(6) PE_DD=PE_Ss=(BiINrob,EXsen)

### 2.2. Building a DT

**Figure 1:**
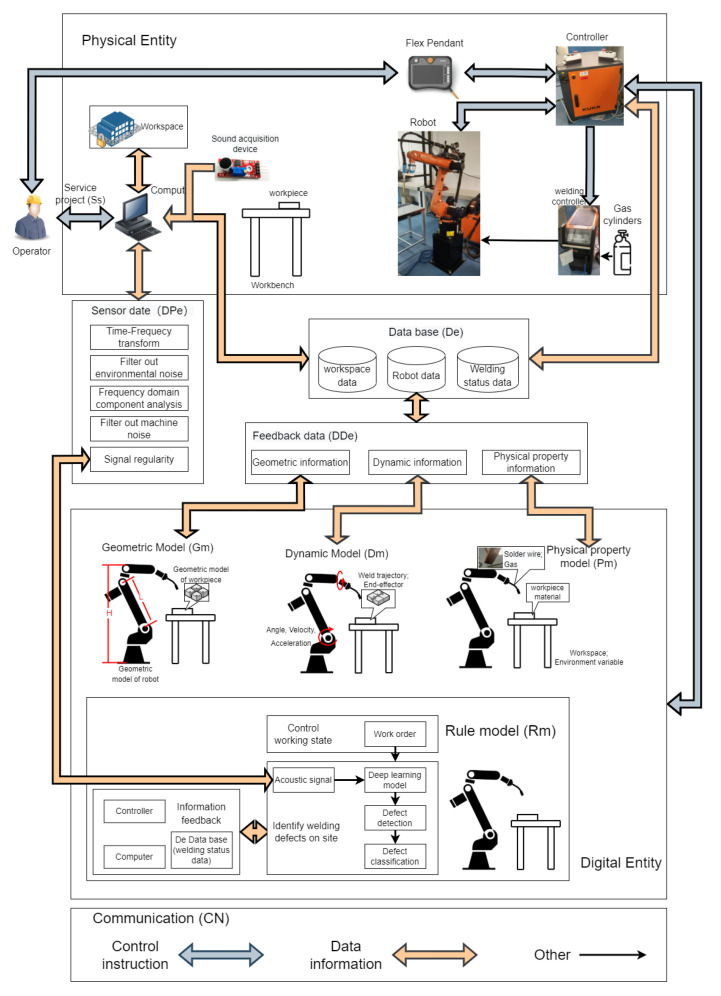
Digital twin system.

As seen in Figure 1, the digital twin system in the experiment was built using the 5D digital twin modeling technique. This paper includes a detailed description of the essential tools and setup for this experiment in the physical entity section. The workspace information was also reflected in the system, because the digital twin system needs to take into account more thorough physical entities. The operator gives the first instructions as the service terminal. Three categories of data—workspace data (environmental data), robot data, and weld status data—are used to organize the data in the database. Robot data mostly refer to the data that the robot itself naturally possesses, such as motion status, geometric details, etc. Weld status data, which primarily record weld quality, weld flaws, and weld types, represent the weld field status fed back through the rule model. To improve data accuracy, the sensor data must go through several filtering processes, which are fully discussed in Section 3. The three primary kinds of digital entity models are geometric models of robots, dynamic models, and physical property models, each of which is independently modeled and returns the necessary data. Regarding the rule model, Section 4. provides a detailed introduction to weld status recognition and weld defect classification.

## 3. Signal Preprocessing

### 3.1. Acoustic Signal Analysis

**Figure 2:**
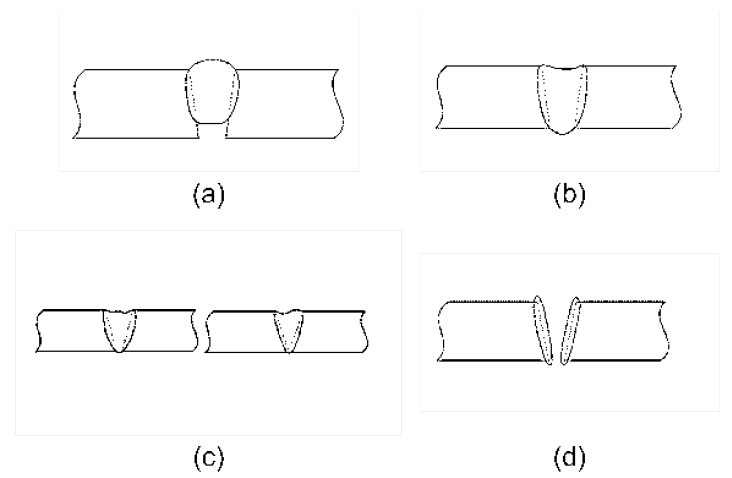
Welding defects: (**a**) incomplete penetration; (**b**) excessive penetration; (**c**) dent; (**d**) burn-through.

In welding research, there are many different sorts of defects, such as cold cracks, hot cracks, incomplete fusion, incomplete penetration, burn-through, blowholes, excessive penetration, dents, and so forth. Some defects may occur after welding, for example, cold cracks may occur in the cooling stage after welding. Some defects occur during welding, such as incomplete penetration, burn-through, collapse, and pits. Figure 2a shows a root that has not completely entered, which is characteristic of incomplete penetration. The workpiece break angle, low current, excessive speed, and inappropriate welding wire angle could all be contributing factors. Incomplete penetration can easily lead to a buildup of stress at the root, which can result in fissures and structural harm. Excessive weld penetration is depicted in Figure 2b, in which the front of the weld is collapsed and the back is lifted. This is the result of excessive workpiece clearance or current, or excessive metal penetration through the rear. Local low-lying areas that occur on the weld surface define the pit shape. The working section of the weld becomes smaller because of pits. The most significant occurrence is burn-through. Molten metal pours out of the groove to create the perforation, as depicted in Figure 2d. This happens because the workpiece is typically overheated, the welding current is too high, and the welding pace is too sluggish. Generally speaking, welding current, the production of these faults, voltage, and speed are connected.

**Figure 3:**
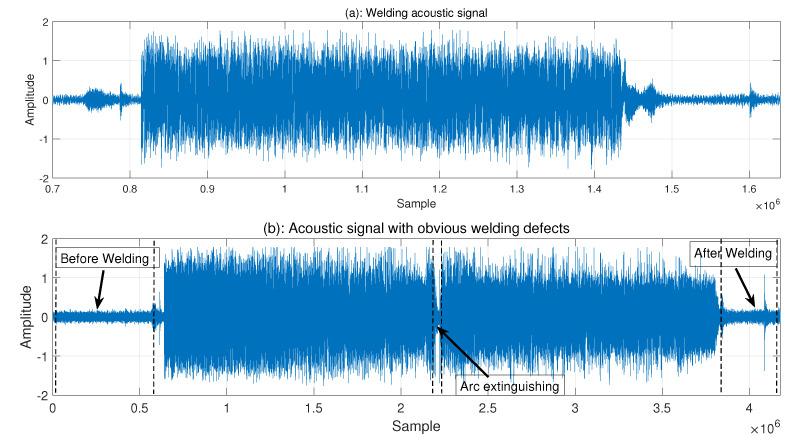
Welding acoustic signals: (**a**) signals with obvious welding defects; (**b**) signals without obvious welding defects.

**Figure 4:**
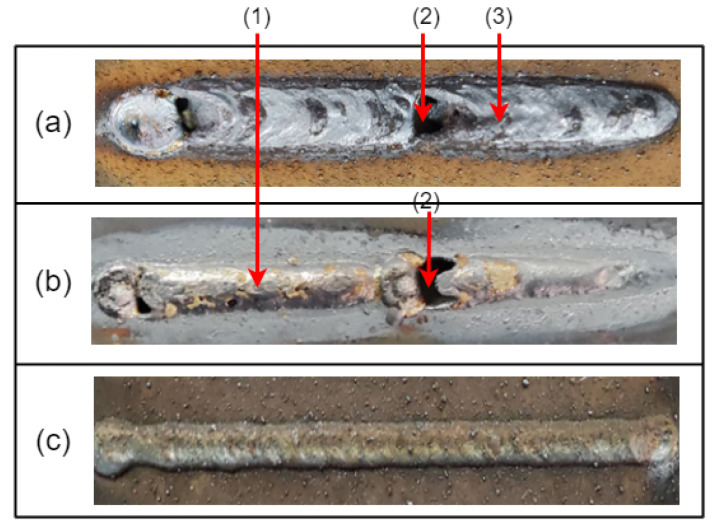
Weld images: (**a**) Front face of weld with obvious welding defects; (**b**) back side of weld with obvious welding defects; (**c**) welds without obvious welding defects. (**1**) Excessive penetration, (**2**) burn-through, and (**3**) dent.

The welding robot workspace in this experiment was an enclosed space. Possible sources of noise in the workspace included the two fans that were there. Second, there was noise generated by the robot movement, as well as some silent noise makers in order to lessen how noise affected the acoustic signals. Both time-domain and frequency-domain analyses of two groups of acoustic sounds were performed in this study. In Figure 3, there are two groups of acoustic signals: those with apparent weld defects and those with good weld quality. Comparable pictures of real welds are shown in Figure 4.

Figure 3a matches Figure 4a,b, while Figure 3b matches Figure 4c. The acoustic signals in Figure 3 have sections of signal with reduced amplitude at the front and back, which represent the situations before and after welding, respectively. At this time, machine noise and outside noise make up the majority of the signal.

It should be noticed that the amplitude abruptly reduces in the middle of Figure 3a, which is the result of the arc extinguishing phenomenon caused by the arc burning through the workpiece and the arc touching the base plate. Indicated by mark (2) in Figure 4, the burnt-through hole is visible. The front and back images of the weld are shown in Figure 4 as (a) and (b), respectively. Combining the two images reveals a range of different weld flaws.

**Figure 5:**
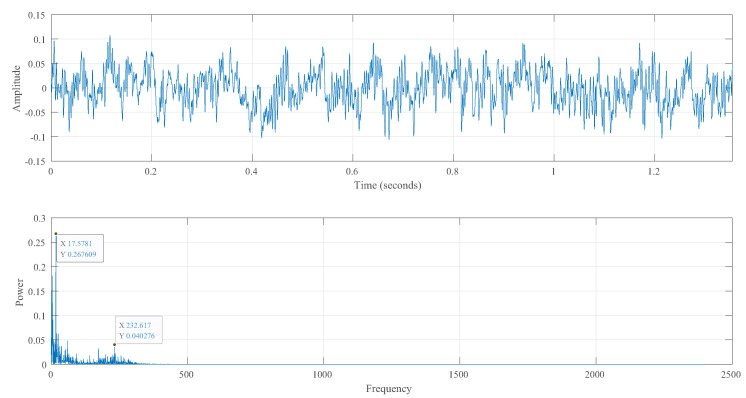
Time- and frequency-domain diagrams of environmental noise.

Figure 3 makes it abundantly evident that there are some irregular peaks and relatively small amplitude variations in the signal time-domain characteristics of the pre-welding and post-welding stages. The machine internal noise and the fan background noise were the main sources of noise in the enclosed environment, as already mentioned. The portion of background noise was collected separately in the shutdown stage, as shown in Figure 5.

**Figure 6:**
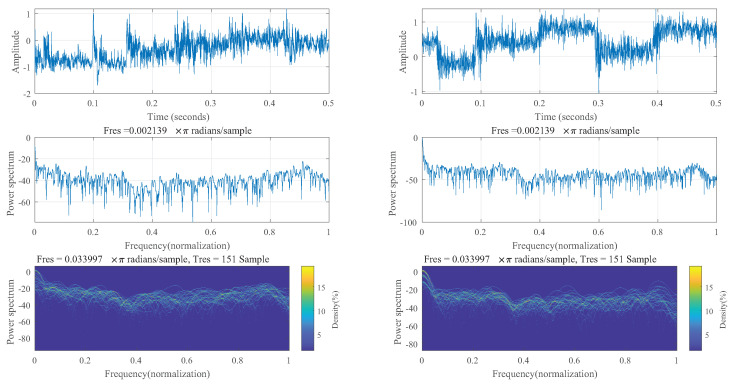
Time domain and frequency domain of two randomly selected welding signals (First row: Time-domain diagram; Second row: Normalised frequency spectrum; Third row: Normalised persistent frequency spectrum.

The amplitude did not exceed 0.1, and the energy was primarily concentrated below the frequency of 200 Hz, characteristic of environmental and fan noise, respectively. In order to separate the machine noise, we extracted 0.5 S data from the signals at both ends of Figure 2 and obtained the time-domain diagram, frequency spectrum, and persistent frequency spectrum of the signal, as shown in Figure 6. The persistent frequency spectrum clearly shows the changes in signal components. In the persistent frequency spectrum, it can be observed that there is an obvious disturbance between the normalized frequencies of 0.4 and 0.6.

### 3.2. Improved Wavelet Denoising

Figure 6 shows that machine noise, which was the major noise source, strongly correlated with weld acoustic signals and could only be seen in certain frequency bands. If only this frequency band were filtered, it would cause serious signal loss. Therefore, this research proposes a wavelet denoising method with an improved threshold function to lessen the interference of machine noise.

(7) $$\left\{\begin{array}{cc} C_{j+1}\;\left(n\right)=\sum kC_{j}\left(k\right)h\left(k-2n\right), & \\ W_{j+1}\left(n\right)=\sum kW_{j}\left(k\right)g\left(k-2n\right), \end{array}\right.$$

The above is the wavelet decomposition of the signal, where $C_{j}\left(n\right)$ are scale factors and $W_{j}\left(n\right)$ are wavelet coefficients.

The traditional threshold functions have a hard threshold and soft threshold, as shown in Formula (8) and Formula (9), respectively.

(8) $$W_{t}=\left\{\begin{array}{ccc} W & \; & \left|W\right|\geq t\\ 0 & \; & \left|W\right|<t \end{array}\right.$$

(9) $$W_{t}=\left\{\begin{array}{ccc} sign\left(W\right)\left(\left|W\right|-t\right) & \; & \left|W\right|\geq t\\ 0 & \; & \left|W\right|<t \end{array}\right.$$

where $W_{t}$ is the wavelet coefficient after the application of the threshold.

The threshold is set to

(10) $$t=\sigma \sqrt{2\ln N}$$

where σ is the standard variance of noise, *N* is the length of the signal. However, hard thresholds and soft thresholds have their own shortcomings. The hard threshold is discontinuous at $t=\left|W\right|$. The soft threshold may be over-compressed when $t\leq \left|W\right|$.

In fact, the energy of arc signals is relatively high and concentrated, and excessive compression affects signal recovery. Therefore, based on the threshold value, we set coefficient A as shown in Formula (11).

(11) $$A=\frac{1}{Z^{\left(t-\left|W\right|\right)}}$$

(12) $$W_{t}=\left\{\begin{array}{ccc} sign\left(W\right)\left(\left|W\right|-At\right) & \; & \left|W\right|\geq t\\ 0 & \; & \left|W\right|<t \end{array}\right.$$

where 0<Z<1 when $\left|W\right|\geq t$, A<1. Therefore, the improved threshold is always greater than the soft threshold, which means that the value of *Z* can be appropriately adjusted according to the signal.

Reconstruction is the reverse process of decomposition. The formula is as follows:

(13) $$C_{j}\left(n\right)=\sum k\left[C_{j+1}\left(k\right)h\left(k-2n\right)+W_{j+1}\left(k\right)g\left(k-2\right)\right]$$

**Figure 7:**
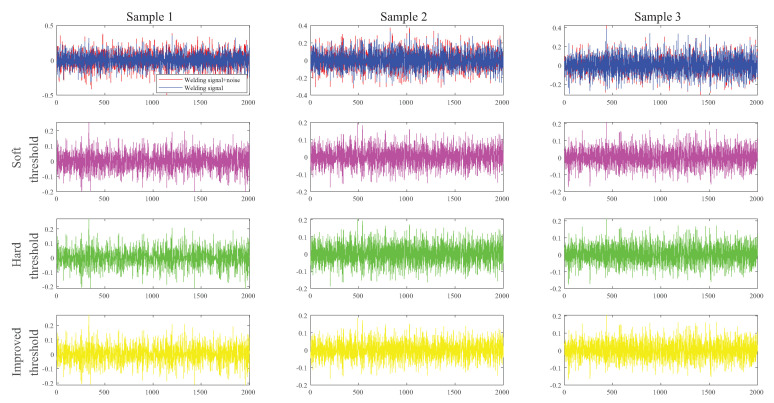
Comparison of wavelet denoising results for three random samples with different signal-to-noise ratios (Row 1: original signal; Row 2: soft threshold wavelet filtering; Row 3: Hard threshold wavelet filtering; Row 4: Improved valence wavelet filtering).

This study compared three groups of various sample signals to demonstrate the efficacy of the wavelet technique, as shown in Figure 7. The test sample signal information was taken from the website SC.chinaz.com. Noise signals of varying decibel levels were added to the three groups of sample signals. The sample signals were subjected to soft threshold-, hard threshold-, and improved threshold-based filtering using the Haar wavelet basis function. The method described in this paper performed well in terms of the noise reduction effect compared with the signal-to-noise ratio (SNR) of the processing results, as shown in Table 2.

**Figure 8:**
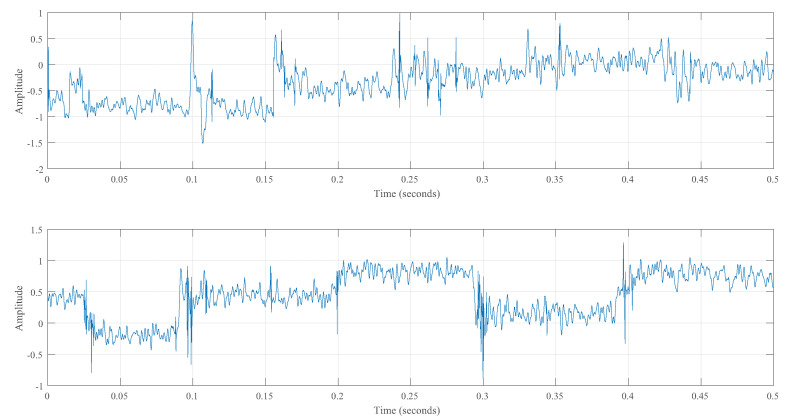
Improved wavelet denoising results for two actual welding signals.

The measured signal was decomposed using a three-layer wavelet, and the signal was denoised as shown in Figure 8.

## 4. Identification and Classification

### 4.1. Classification Model

The usual sequential signal, the acoustic signal, has a very high degree of continuity. In order to identify and categorize the information reflected by acoustic signals, in this work, we developed an SeCNN-LSTM depth learning model.

The discipline of speech recognition has extensively explored acoustic signal processing. However, although they can be used as a guide, related voice recognition technologies are not entirely relevant, because signals from human speech are the primary focus of speech recognition. For instance, the widely used MFCC (mel-frequency cepstral coefficient) feature classification approach enhances the human auditory frequency range while suppressing the high-frequency band [26,27]. Suppressing high-frequency signals results in a significant loss of information when industrial machinery is used as the research object.

There are currently three established techniques for identifying auditory signals. First, the MFCC approach is represented by cepstral feature extraction and classification [28]. For example, a signal of 16 KHZ usually intercepts a frame of the cepstrum, with a 39D channel output. Cepstral feature extraction also makes use of LPCCs (linear prediction cepstral coefficients), LSFs (line spectral frequencies), PLP (perceptual linear prediction), and other algorithms [29,30]. Additionally, filter banks that have an output of up to 80D channels are used to extract the features. The alternative is to sample and categorize signals directly. The benefit of this is that the output channel can be altered to prevent catastrophes caused by high and small numbers of dimensions.

**Figure 9:**
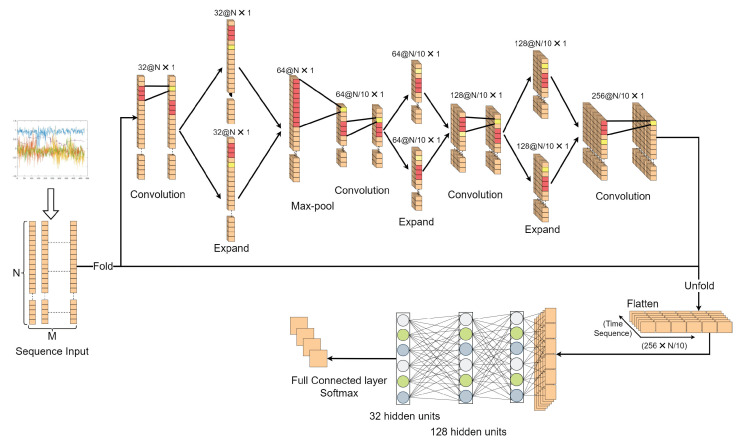
SeCNN-LSTM structure diagram (**N**): Number of sampling points per group (**M**): Number of data groups.

Figure 9 displays the deep learning model created in this paper. The experimental requirements for the deep learning model include a lightweight design in addition to an effective recognition rate, because a certain level of portability is necessary for the creation of digital twin systems. As a result, we built the one-dimensional convolution layer feature extraction process as a three-layer parallel extended structure. Although it resembles the traditional Squeeze network, the parallel convolution kernel size is the same. Data are folded for one-dimensional convolution when they are input and then expanded, and their time series are regressed. The output timing signal group has 256 channels. Finally, the two layers are LSTM (Long Short-Term Memory) structures of 128 and 32 hidden units.

### 4.2. Model Training and Parameters

Dataset: The sampling frequency of the acoustic signal was 4.8 KHz, so 0.1 S signals were intercepted as a group, where each group contained 480 sampling points. A total of 90 groups were intercepted upon the occurrence of each defect type. Because the burn-through signal had a certain degree of instantaneity and abruptness, the burn-through signal was 40 groups. The data for this experiment is 310 timing signals and the amount of data is 148,800. 80% is the training set and 20% is the testing set.

Loss function: Softmax is often applied to multi-category tasks, also known as multi-category categorical cross entropy loss.

(14) $$S\left(x,y\right)=-\sum_{i=1}^{N}q\left(x_{i}\right)\log q(x_{i})$$

(15) $$q\left(x_{i}\right)=yic_{i}\;i\in \left[1,N\right]$$

where $c_{i}$ is $x_{i}$ and corresponds to the target class.

Activation function: In the one-dimensional convolution part of the model, Formula (16) is used as the activation function.

(16) $$Ruel\left(x\right)=max\left(0,x\right)=\left\{\begin{array}{c} 0,x<0\\ x,x>=0 \end{array}\right.$$

LSTM performs well in current time series data classification and prediction tasks. The saturated activation function is more suitable for the requirements of LSTM. Formula (17) is used as the gate activation function for the forgetting gate, input gate, and output gate. When generating candidate memory, the state activation function is Formula (18).

(17) $$Sigmoid\left(x\right)=\frac{1}{1+e^{-x}}$$

(18) $$Tanh\left(x\right)=\frac{e^{x}-e^{-x}}{e^{x}+e^{-x}}$$

Weight initialization: In the training process, weight has a significant impact on the effect of network training. The optimal scheme is obtained by comparing different weight initialization functions. The ”Glorot” initializer independently samples from a uniform distribution with 0 and weights $W_{g}$.

(19) $$W_{g}∼U\left[-\frac{1}{\sqrt{n}},\frac{1}{\sqrt{n}}\right]$$

where $U\left[-\frac{1}{\sqrt{n}},\frac{1}{\sqrt{n}}\right]$ is the uniform distribution in interval $\left[-\frac{1}{\sqrt{n}},\frac{1}{\sqrt{n}}\right]$ and *n* is the size of the previous layer. The "He" initializer independently samples from a uniform distribution with 0 and weights $W_{h}$.

(20) $$W_{h}∼U\left[0,\sqrt{\frac{2}{Vn_{i}}}\right]$$

where $Vn_{i}$ indicates the current input size. The “Orthogonal” initializer refers to the orthogonal matrix decomposed from the random matrix sampled from the normal distribution. “Narrow normal” refers to sampling from 0 to 0.01 standard deviation.

**Figure 10:**
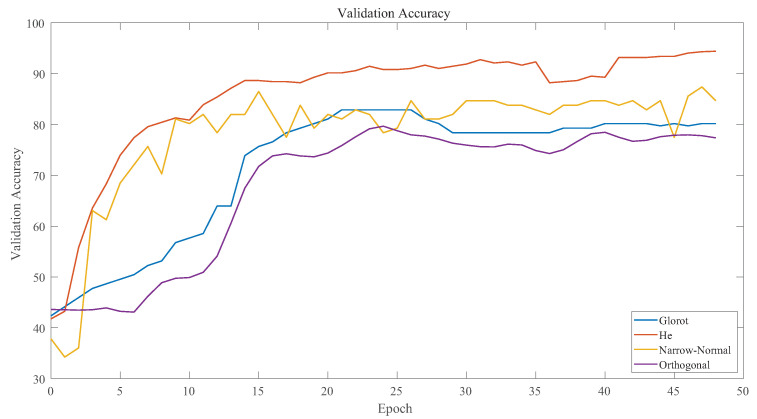
Weight initializer comparison.

Figure 10 shows the training results under the influence of different initialization weight functions. From the figure, it can be seen that the training result of the "HE" initialization function was the best one.

The maximum iteration was 100, and the learning rate was 0.005.

**Figure 11:**
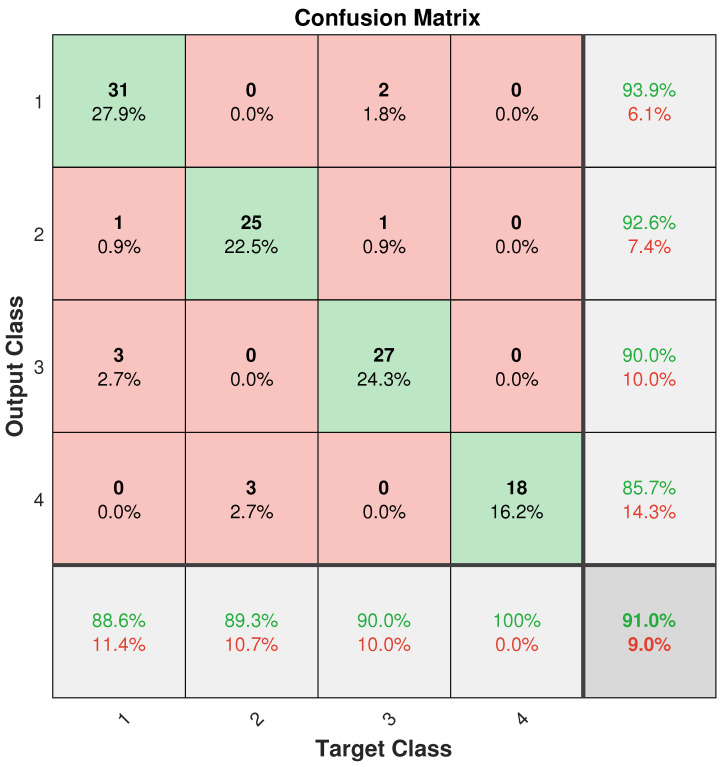
Test confusion matrix.

The SeCNN-LSTM network was trained as shown in Figure 11. Target class refers to the prediction classification item, while output class denotes the true classification item. The sample was unbalanced because of the experimental circumstances, as was indicated in the preceding article. Additionally, since a random sample among all the samples was used to create the test set data, each sample in the confusion matrix was likewise out of balance.

(21) $$Acc=\frac{tr(con)}{Sum(con)}$$

where Acc refers to accuracy; tr(con) refers to the trace of the confusion matrix, that is, the sum of the main diagonal elements; and Sum(con) is the sum of all elements in the matrix. The test set confusion matrix verification accuracy reached 91.0%.

### 4.3. Model Comparison

In order to verify the superiority of the model described in this paper, the SeCNN-LSTM model was compared with other seven models using multiple indicators. Models LSTM, BiLSTM(Bidirectional Long Short-Term Memory), CNN-LSTM, CNN-BiLSTM, GRU(Gated Recurrent Units), CNN-GRU, and CNN-SVM were compared. In the area of sequence classification, the widely applied and developed deep learning models LSTM and BiLSTM were used. BiLSTM is composed of two LSTMs, with one receiving inputs forwards and the other backwards. Another type of RNN is GRU. GRU is better in line with the demands of processing power and time cost than LSTM and has fewer parameters. The goal of this paper was to demonstrate the benefits of the deep learning model described in this paper in one-dimensional sequential classification networks. The deep learning model in this paper is a CNN-RNN structure, and the majority of the models involved in the comparison are also CNN-RNN structures.

**Figure 12:**
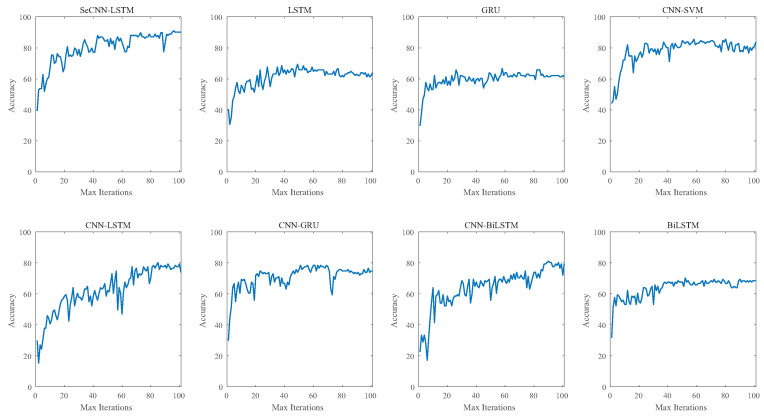
Test accuracy curve.

The test set accuracy of each model is shown in Figure 12. The test accuracy of the SeCNN-LSTM model used in this study was 90.99% after 100 iterations, while those of CNN-SVM and CNN-BiLSTM were 83.78% and 78.28%, respectively. The test accuracy values of other models were under 80%.

In binary classification models, precision and recall are commonly employed as important metrics to check the model classification outcomes. This idea is expanded in the multi-classification model to cover the classification of each sample separately. A metric used to describe the outcomes of predictions is precision. It speaks of the likelihood of a real positive sample among all anticipated positive samples. The initial sample is described by the recall rate. It is the likelihood that the expected positive sample will be positive.

As can be seen in Table 3, the SeCNN-LSTM model classification precision was significantly higher than those of other classification models, with the first three samples being classified correctly with more than 90% precision and the fourth sample being classified correctly with over 85% precision.

Each sample in the SeCNN-LSTM model had a recall rate of over 88%, which is significantly greater than those of conventional classification models.

In actual classification, sample imbalance is inevitable. ROC (receiver operating characteristic) and AUC (area under the curve) indicators can ignore sample imbalance to assess the classification and prediction abilities of a model.

**Figure 13:**
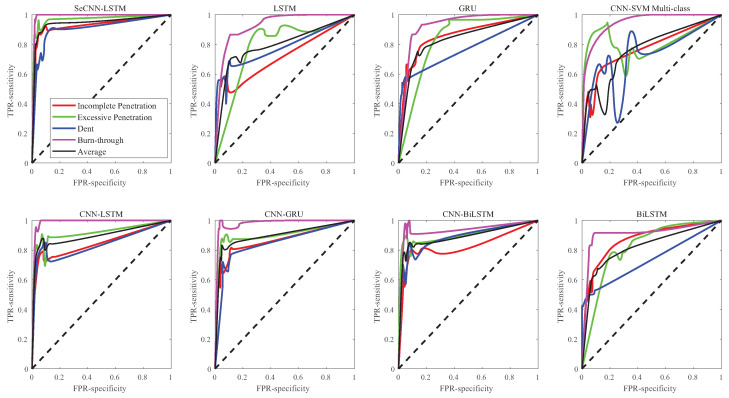
ROC curve comparison.

ROC refers to the relationship between sensitivity (recall) and specificity under different classification thresholds. ROC is a concept based on a binary classification model. In a multivariate classification model, each sample needs to be considered separately. As shown in Figure 13, the ROC curves of the eight classification models are compared. It could be observed that the ROC curve of the SeCNN-LSTM model is smoother and more concentrated in the (0,1) coordinates.

AUC refers to the area values enclosed by ROC curve and coordinates. The closer the AUC value is to 1, the better the model is. When the AUC value is less than 0.5, the model is invalid. It can be seen from Table 4 that the AUC values of all samples of the SECNN-LSTM model are above 0.9, much higher than other models.

**Figure 14:**
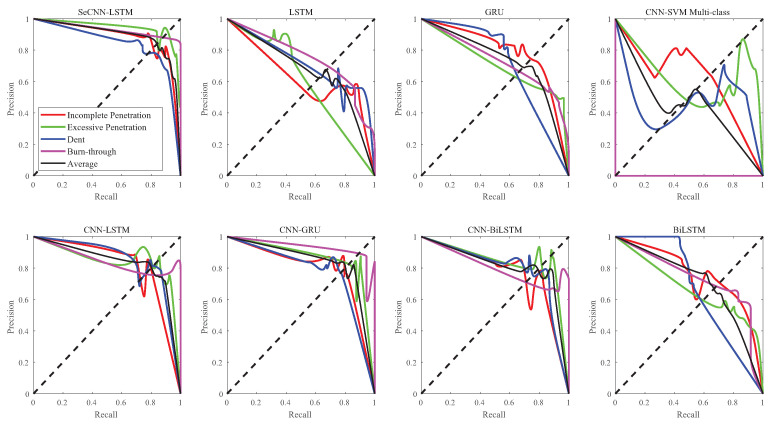
P–R curve comparison.

As shown in Figure 14, the P–R curve refers to the relationship between precision and recall under different classification thresholds. Precision and recall are a pair of mutually exclusive indicators. In an actual sample, the highest accuracy rate and recall rate cannot be obtained at the same time, so only an ideal balance value can be obtained. It can be observed from the P–R curves of the eight models that the curves of SeCNN-LSTM model are smoother and more concentrated in the (1,1) coordinates.

The area values enclosed by the P–R curves (P–R AUCs) are shown in Table 5. The value of each sample of the SeCNN-LSTM model is above 0.83.

The F1-score indicates the best balance between precision and recall. As shown in Table 6, comparing the F1-score of each sample of the eight models, the model described in this paper showed the best performance.

In summary, we compared the eight different models’ accuracy, precision, recall, AUC, ROC, F1 score and other measures. The findings demonstrate that the model presented in this research outperforms the one-dimensional sequence data classification model in terms of performance, stability, and sample balance.

## 5. Conclusions

This research established a digital twin system for robot weld field detection based on acoustic signal processing and acoustic signal analysis. The identification and classification of auditory signals was performed using an enhanced SeCNN-LSTM deep learning model. The digital twin system incorporates both a deep learning model and a signal processing technique.

The digital twin system modeled in this paper is insufficient. First, the modeling of rule models was covered at length in this study, but the modeling methods of the physical material model and the dynamic model in the digital twin system were not discussed in detail. Second, the deep learning model discussed in this research was found to have 91% verification accuracy, which is not the best possible outcome, meaning that there is still room for development.

Weld field inspection is both a research focus and a challenge in the welding industry, due to the complicated on-site environment, influencing factors, non-linear welding conditions, and other considerations. Despite having solid timing, the acoustic signal is susceptible to interference. Therefore, future research could focus on multi-sensor or multi-modal weld information detection to reflect the welding conditions using several types of weld information.

## Figures and Tables

**Table 1:** Characteristics of materials of physical equipment.

	Name/Brand	Material
Workpiece	Stainless steel S30400	C=0.07, Mn=2, P=0.045, S=0.03, Si=0.75, Cr= 17.5–19.5, Ni= 8–10.5
Solder Wire	ER70s-6	C≤0.08, Si≤1.15, Mn= 1.4–1.85, P≤0.025, S≤0.035, Cu≤0.5
Shielding Gas	CO_2_	

**Table 2:** Signal-to-noise ratio (SNR).

	Samle 1	Sample 2	Sample 3
Soft shreshold	3.6525	6.5828	12.2800
Hart shreshold	3.5645	6.3638	12.1649
Improved shreshold	4.1886	6.9739	12.3590

**Table 3:** Accuracy, precision and recall comparison.

	Accuracy	Precision				Recall			
		Incomplete Penetration	Excessive Pentration	Dent	Burn-Through	Incomplete Penetration	Excessive Pentration	Dent	Burn-Through
SeCNN-LSTM	90.99%	93.93%	92.59%	90%	85.71%	88.57%	89.28%	90%	100%
LSTM	63.96%	93.3%	47.3%	93.8%	64%	38.9%	86.7%	53.6%	94.1%
GRU	61.26%	67.9%	61.9%	85.7%	66.87%	59.4%	83.9%	42.9%	90 %
CNN-SVM	83.78%	71.4%	92.3%	73.1%	0%	74.1%	88.9%	70.4%	0%
CNN-LSTM	73.87%	89.3%	73.5%	68.6%	92.9%	61%	96.2%	88.9%	76.5%
CNN-GRU	74.77%	72.7%	83.8%	83.3%	75%	88.9%	91.2%	50%	81.8%
CNN-BiLSTM	79.28%	64.7%	84.4%	88.9%	72.2%	81.5%	84.4%	63.2%	92.9%
BiLSTM	68.47%	86.7%	58.8%	78.3%	68.2%	39.4%	90.9%	62.1%	93.8%

**Table 4:** AUC comparison.

	Incomplete Penetration	Excessive Pentration	Dent	Burn-Through	Average
SeCNN-LSTM	0.9274	0.9687	0.9052	0.9895	0.9443
LSTM	0.7172	0.7827	0.7809	0.9279	0.7952
GRU	0.8573	0.8343	0.7664	0.9269	0.8320
CNN-SVM	0.7687	0.8188	0.7241	0.9470	0.7634
CNN-LSTM	0.8426	0.9159	0.8353	0.9868	0.8951
CNN-GRU	0.8641	0.9012	0.8448	0.9795	0.8965
CNN-BiLSTM	0.8238	0.9016	0.8842	0.9310	0.8872
BiLSTM	0.8695	0.8203	0.7346	0.9097	0.8210

**Table 5:** P–R AUC comparison.

	Incomplete Penetration	Excessive Pentration	Dent	Burn-Through	Average
SeCNN-LSTM	0.8838	0.9428	0.8391	0.9322	0.8938
LSTM	0.6256	0.5991	0.7160	0.7461	0.6719
GRU	0.7900	0.7061	0.7103	0.7323	0.7303
CNN-SVM	0.6125	0.6317	0.4665	0	0.4501
CNN-LSTM	0.8154	0.8401	0.8479	0.8492	0.8323
CNN-GRU	0.7955	0.8612	0.7763	0.9329	0.8358
CNN-BiLSTM	0.7696	0.8338	0.7951	0.8070	0.8061
BiLSTM	0.7546	0.6678	0.6683	0.7284	0.7018

**Table 6:** F1 score comparison.

	Incomplete Penetration	Excessive Pentration	Dent	Burn-Through	Average
SeCNN-LSTM	0.7406	0.7741	0.6984	0.7561	0.7436
LSTM	0.5628	0.4454	0.5911	0.5414	0.5716
GRU	0.6345	0.5556	0.5857	0.5672	0.6212
CNN-SVM	0.5023	0.6321	0.5384	0	0.3984
CNN-LSTM	0.6818	0.7488	0.7166	0.7199	0.7212
CNN-GRU	0.6945	0.7573	0.6685	0.7871	0.7296
CNN-BiLSTM	0.6441	0.7720	0.6766	0.7387	0.7127
BiLSTM	0.5652	0.5488	0.5325	0.5631	0.5792

## Data Availability

The data that support the findings of this study are available from the authors upon reasonable request.
